# Daily staffing trends and variation in Swiss long-term care from 2018–2023: A retrospective longitudinal analysis

**DOI:** 10.1016/j.ijnsa.2025.100395

**Published:** 2025-07-29

**Authors:** Catherine Blatter, Michael Simon, Franziska Zúñiga

**Affiliations:** Institute of Nursing Science, Department Public Health, Medical Faculty, University of Basel, Switzerland

**Keywords:** Health care facilities workforce and services, Health personnel, Health workforce, Long-term care, Nursing homes, Routinely collected health data

## Abstract

**Background:**

Workforce shortages present an ongoing challenge for long-term care service providers in Switzerland. After the COVID-19 pandemic, there is an urgent need to understand current workforce trends to strengthen the Swiss long-term care setting.

**Objective:**

We aimed to describe pre-, peri‑, and post-pandemic trends and variation of long-term care staffing.

**Design:**

We conducted a retrospective longitudinal analysis using daily administrative routine data from 2018 to 2023.

**Setting(s):**

A multisite long-term care facility in Switzerland with 180 beds.

**Methods:**

We applied a time-series decomposition – a method to distinguish trends from seasonal effects and residual variation – to describe trends and variation for three outcomes: a) the supply-demand-match (i.e., how did the available direct staff cover the actual care demand), b) the number of full-time absences (i.e., how many staff members were scheduled to work but were absent), and c) the number of temporary staff from an internal pool or external agency. We used means, confidence intervals, and percentages to summarize the yearly averages.

**Results:**

We linked data from 533,003 staff shifts and 387,585 resident days across the 6 study years. Overall, we observed constant variation but a decreasing trend of supply-demand match from a daily average number of staff of +6.88 [95% confidence intervals CI 6.86 - 6.90] in 2018 to -0.23 [95% CI -0.24 – -0.22] in 2023, meaning that for each year of the study, there was roughly one fewer staff member available per day for the same resident case mix. Simultaneously, daily staff absences increased from an average of 11.08 [95% CI 11.06 - 11.09] to 14.23 [95% CI 14.19- 14.27]. Absences decreased at the beginning of the pandemic (2020) but continuously increased from 2021 onwards, especially those with a duration of ≥ 1 week. As an organizational response, the number of shifts worked by temporary staff has increased from 5.5% in 2018 to 17.7% in 2023.

**Conclusions:**

We found a constant variation but a clinically noticeable downward trend of supply-demand-match from pre- to post-pandemic, largely driven by staff absences. It could not be reversed despite an increased deployment of temporary staff from both an internal pool and external agencies. We have revealed profound effects of the pandemic on an organization’s ability to meet the required care demand. Healthcare policy should consider alternative reimbursement strategies to alleviate the financial burden associated with a high number of absences.

**Registration:**

not registered

**What is already known**

•Workforce shortages present ongoing challenges for long-term care, in Switzerland and other nations

•After the pandemic, there is an urgent need to understand current staffing trends to strengthen the long-term care setting

Alt-text: Unlabelled box

**What this paper adds**

•We have confirmed a downward trend of available staffing after the pandemic, influenced by a substantial increase of long-term staff absences

•The trend could not be reversed despite a consequent rise in reliance on temporary staff

•We have underscored the post-pandemic impact on organizational capacity to meet care demands and individual staff workload

Alt-text: Unlabelled box

## 1 Background

Long-term care facilities play a crucial role in the health care system by providing essential services to an aging population with complex care needs. In Switzerland, as in many other countries, the demand for residential long-term care services, such as nursing homes, is escalating due to demographic shifts, including a growing older population and longer life expectancies (Lussi et al., 2024). According to the Swiss Federal Office of Public Health, by 2040 an additional 54,000 places in long-term care facilities will be required, which translates to +150 % of the current availability (Pellegrini et al., 2022). Furthermore, individual resident acuity has increased in recent years in Switzerland (Federal Statistical Office FSO; University of Neuchâtel UNINE; University of Fribourg UNIFR; Swiss Academy of Humanities and Social Sciences SAHS, 2024). This rapidly rising demand, both in volume and complexity, places considerable pressure on long-term care facilities to maintain optimal staffing levels to ensure high-quality care and resident safety.

Staffing levels in long-term care facilities are critical for ensuring optimal resident care (Mukamel et al., 2023). Research indicates that higher staffing levels, particularly for registered nurses (RNs), are associated with favorable resident outcomes, such as fewer pressure ulcers, lower rates of infections, and improved overall health status (Clemens et al., 2021; Mukamel et al., 2024). Nonetheless, achieving and sustaining optimal staffing levels and staff mix– that is, the combination of staff with different educational backgrounds, such as RNs, licensed practical nurses (LPNs), or certified nursing assistants (CNAs)–remains challenging due to various factors, including workforce shortages, financial constraints, regulatory requirements, and volatile care demand (Griffiths et al., 2020). To ensure at least an adequate number of care staff is available despite these challenges, many countries have implemented minimum staffing standards (Harrington et al., 2012). However, even with these standards, long-term care facilities in the U.S. failed to meet these daily staffing expectations in more than 50 % of cases, according to a payroll-based analysis from the US (Geng et al., 2019).

In Switzerland, minimum staffing standards for staffing levels are not publicly disclosed, although they are used by authorities to certify long-term care organizations. Recommendations regarding staff mix are publicly reported for certain cantons (i.e., federal units forming the Swiss confederation), but these vary considerably, with minimum recommendation for RNs ranging from 5 % to 28 % (ARTISET and CURAVIVA, 2022) without accounting for resident case mix. The Swiss Federal Statistical Office publicly reports annual structural staffing levels (i.e., full-time equivalents of employees), but more detailed figures (i.e., monthly or weekly) or operational staffing levels (i.e., akin to payroll-based journals in the US) are not available for Switzerland. Moreover, the publicly reported annual staffing levels do not offer a direct comparison with contemporaneously measured resident demand.

The challenge to maintain an adequate care workforce in long-term care, particularly in the aftermath of the pandemic (McGilton et al., 2020), necessitates a comprehensive understanding of current staffing trends, including those in Switzerland. In the absence of published staffing regulations, publicly available operational staffing data, or previous granular perspectives on staffing, our study aimed to address this gap by describing granular trends and variation in daily care staffing patterns before, during, and after the pandemic in Switzerland.

## 2 Methods

### 2.1 Study design and setting

We conducted a retrospective, descriptive, longitudinal analysis of routinely collected data within a publicly funded multisite long-term care organization in Switzerland. At of the end of 2022, there were 1485 officially registered long-term care facilities in Switzerland, with an average size of 67.6 beds (Federal statistic on Nursing Homes [WWW Document]). Our study center is a multisite long-term care organization comprising of 250 beds, offering both short- and long-term care, as well assisted living facilities. This study considered data from 7 units across two locations, which provide 24-hour/day long-term care for up to 180 residents. Of these 7 units, 4 specialize in care for residents living with dementia including palliative care as well as psychological pathologies. The remaining three units provide care to residents, where dementia is not the main reason for admission, including palliative care, rehabilitation (including extended short-stay residents after hospitalization), and specialized care for neurological diseases. As evident from the size and the diversity of services offered, the long-term care organization is a strongly positioned long-term care provider in Switzerland. During the pandemic, the nursing home temporarily opened an (additional) designated cohort unit for residents with acute COVID-19 infection, accessible to both current residents and residents from other organizations. For our analysis, we excluded the designated COVID-19-unit.

Our study period included daily data across six years, from January 1, 2018 up to December 31, 2023. The timeframe allowed for a natural incorporation of the pandemic whilst providing up-to-date data.

### 2.2 Participants

#### 2.2.1 Resident sample

We included data from all residents admitted to any of the included units during the study period. No restrictions were applied regarding the type or length of their stay.

#### 2.2.2 Nursing home care staff sample

We included data from nursing and care staff members who worked at least one direct or indirect care shift in the included units during the study period. Most staff members are trained as nursing care personnel; however, several units incorporate therapy and activation staff as fixed parts of their teams. To address the unequal distribution of these groups and to achieve a sufficiently large group size, we established three distinct staff groups for further analysis based on clinical experience and scope of practice with the following reattribution: 1) registered nurses (RNs), including diploma- educated RNs with a 3–4 year education, bachelor's or master's degree educated RNs and physiotherapists; 2) licensed practical nurses and care staff (LPNs) with a 3-year education with a federal certificate, including activation/music/art therapists; 3) certified nursing assistants (CNAs) with a 2-year education or on the job training, including students and apprentices from groups 1) −3). Unlike many other countries, in Switzerland students and apprentices are employed by and regularly work in the organizations throughout their education. To account for their limited experience and in accordance with cantonal staffing recommendations we reduced the working time of students to 25 %. To enable international comparison, where students are rarely considered in analyses, we categorized them within the lowest staff group (CNAs). The number of nursing students remains constant over time, thus not affecting variation in CNA staffing patterns. In further accordance with canton staffing recommendations, we excluded shifts from all other staff (i.e., volunteers, interns staying less than 3 months, accounted for less than 1 % of all shifts) (Pflegeversorgung - Vorlage für die Berechnung des Mindeststellenplans, 2023). Given the inclusion of therapy staff, we use the term “nursing home care staff” instead of nursing staff throughout this study. Irrespective of our study and at the beginning of our follow-up time before COVID-19, the organization implemented a structured absence management system including bonuses and rewards to incentivize attendance and reduce absenteeism.

#### 2.2.3 Context of care delivery

##### 2.2.3.1 Direct care versus indirect care

Staff members are employed by the organization and assigned to specific unit. Each unit is led by a unit supervisor and complemented by a nurse expert (i.e., a nurse in an expanded role (Basinska et al., 2021), along with regular ward staff, which includes three groups: RNs, LPNs and CNAs. Unit supervisors need to hold at least a RN degree and managerial leadership training, and nurse experts need to hold an RN degree and additional education (i.e., at the minimum a bachelor’s degree).

The regular ward staff (RNs, LPNs and CNAs) primarily deliver *direct care*, to which all activities and shifts targeted in direct resident contact, i.e. a typical day shift, late shift or night shift, are counted. Occasionally, both the unit supervisor or the nurse expert are scheduled to work shifts in direct care. Most of the time, the unit supervisor and nurse expert engage in managerial and clinical leadership and supervisory responsibilities without direct resident assignments, called *indirect care*. Yet, both are physically located in an office on the ward, thereby representing surplus personnel accessible in emergencies.

##### 2.2.3.2 Deployment of internal pool and external agency staff

Each employed staff member is attributed to a certain unit, referred to as the focal unit, where they typically provide their shifts. Occasionally, staff members are required to substitute on a different unit within the organization, called foreign unit, consequentially reducing the staff supply on their focal unit. The organization also maintains an internal staff pool. Pool staff members are also employed by the organization, since their focal unit (pool) is never the place where they deliver their work (which is always on a foreign unit), their deployment never reduces supply on any of the focal study units. When all internal resources are exhausted, the organization relies on external agency staff. To assess the number of pool and agency staff working on a unit, we distinguish between staff members from external agencies (external to the organization), staff members from the internal pool (external to the unit, but not reducing the staff supply in another unit) and staff members from another study unit (external to the unit, and reducing staff supply of their focal unit).

### 2.3 Data sources and linkage

We obtained data from five different administrative data sources. Resident information was extracted from the billing and claims system, which provided information on the exact dates of stay for each resident along with the associated care demand level. This information was matched with an export from the administrative system to get sample characteristics such as age and sex. To gather staff data, we obtained information from different databases: timepoints and unit affiliation from the roster planning system was merged with payroll administrative data at the staff level to include information on personal characteristics (age, sex), facility tenure and employment percentage. A diagram illustrating all data sources and linkages is shown in Supplementary Material A.

### 2.4 Data access, cleaning and preparation methods

The first author of the study had access to raw but deidentified exports from both data sources for staff and resident data for the study period from 2018 to 2023. Initial cleaning focused on reducing both datasets to the study population and anonymizing them prior to further analysis. Given the nature of the data sources (roster planning, payroll, and billing systems), all three databases are closely monitored and updated upon completion, indicating a limited need for data cleaning or treatment of missing data. Still several quality checks were performed. Data linkage and loss of participants was documented at each stage, and, where possible, the information was verified between databases. In cases of conflicting information between the databases, the first author validated the information with the IT team at the study site and developed strategies to determine the most reliable data source.

### 2.5 Measurements and variables

#### 2.5.1 Participant samples at baseline

To account for repetitive measurements in the sample, we calculated baseline characteristics for the population, defined as either a valid measurement in 2018 (if the person was already attributed to the study site) or in the case of later attribution, the first available measurement during the study period. The resident sample is described with basic demographic information such as age (years), sex (male, female, according to Swiss law), care complexity at baseline and presence during the study period. The staff sample is described by age, sex, occupational group and organizational tenure (years) at baseline.

#### 2.5.2 Time-varying variables of interest

##### 2.5.2.1 Resident demand

Resident demand is described by *bed occupancy* and *care complexity*. Bed occupancy is calculated as daily resident census relative to the yearly maximum resident census of a unit. Proportional reporting allows for between unit comparison in spite differing unit size. *Care complexity* was operationalized through care demand levels. In Switzerland, each resident is assessed at least every nine months by a resident assessment instrument. At the study site, the Swiss version of the resident assessment instrument RAI NH-Version is used (Anliker and Bartelt, 2015), which classifies resident care needs into one of 36 resource utilization groups. Each resource utility group is attributed to one of twelve care demand levels (a scale from 1–12), where formally, each care level indicates up to 20 min of additional care needed per day. The care demand level is used for reimbursement claims to health insurance. Accordingly, there is a reassessment and -classification each time the resident has a change in the health status that affects the care demand level. We calculate the mean daily care demand level per unit and facility. Furthermore, we translate the *care demand level* into a time measure according to the staffing recommendation of the canton of the study site to achieve a time-based demand-value for direct comparison with staff time.

##### 2.5.2.2 Staff supply

Staff supply is described by *staff time* per day. From roster planning data we extracted the stamped shifts, mapped staff’s presence for every minute during the study timeframe and aggregated this to total staff time in minutes per study day. A full working day of one staff member lasts 504 min (which corresponds to 8.4 h). As this metric is cumbersome to interpret, we transformed the total staff time into staff members working a full day (i.e., calculating total time / 504), to reach a more natural unit of analysis when reporting the results. Several grouped analyses of staff time were applied, e.g., by staff group and or by direct or indirect care. *Staff mix* refers to the daily proportional staff time for each staff group compared to total staff time.

##### 2.5.2.3 Staffing measures

Two outcome measures to assess direct-care staffing were calculated: *hours per resident day* (HPRD) and *supply-demand-match*.

HPRD is a popular international measure used for staffing analyses and regulations in long-term care. The calculation is as follows:

$$HPRD=\frac{\text{Total}\;\text{direct}\;\text{care}\;\text{staff}\;\text{time}\;\left[hours\right]}{Resident\;census}$$

The calculation of HPRD was stratified by staff groups, i.e., RN—HPRD. HPRD does not account for individual resident care complexity.

The second measure, *supply-demand-match* was self-developed and accounts for resident complexity. It is calculated as the daily difference between supply and demand:

$$\text{Supply}-\text{demand}-\text{match}=\;\frac{Total\;direct\;care\;staff\;time\;-total\;care\;demand\;time\;\left[minutes\right]\;}{504}$$

The resulting difference in minutes was reverse transformed into number of staff members working a full day as described above, to reach a more natural unit. A positive number indicates a theoretical (residual) surplus of staff supply; a negative number indicates a theoretical surplus of resident demand. Given the lack of a validated or publicly regulated threshold for sufficient staff in Switzerland, we use the theoretical threshold of values below zero, but urge caution in the interpretation of this value as sufficient staff.

##### 2.5.2.4 Number of absences and deployment of pool or agency staff

From all staff shifts we calculated the daily number of full-time absences, according to entry in the roster planning system. Each absence is classified as either an illness of duration < 1 week, illness ≥ 1 week or an accident. To assess the deployment of pool and agency staff, we calculated the daily number of staff members who are not working on their focal unit (see also description in previous section). We distinguish between external agencies, internal pool and other (internal) study units.

### 2.6 Statistical analyses

To describe the sample populations, we calculated descriptive statistics with measures of central tendency and dispersion for continuous variables, and absolute and relative frequencies for categorical variables. We visualized trends and variation over time and report yearly aggregated values using measures of central tendency (mean, median) and dispersion (95 % CIs, interquartile range).

For our analysis, we applied an additive STL-decomposition which is a method to model trends while accounting for effects in seasonality (Cleveland et al., 1990). The corresponding equation is:

$$y_{t}=S_{t}+T_{t}+R_{t}$$

where y_t_ is the measurement at a given timepoint, S_t_ the seasonal component, T_t_ the trend-cycle component, and R_t_ the remainder component (Hyndman and Athanasopoulos, n.d.). We used a maximal seasonal window of 365 days (natural season in daily data across multiple years) to fit the models. Consequentially, variation is based on operational values y_t_, while trends are based on fitted values for T_t ._

Data preparation and statistical analysis was undertaken with R, version 4.4.2 for Mac OS X (R Core Team, 2024). For data preparation we used the packages arrow (Richardson et al., 2024) and ivs (Vaughan, 2023). For data analysis we used dplyr (Wickham et al., 2023), feasts (O’Hara-Wild et al., 2024), tsibble (Wang et al., 2020); for visualization ggplot2 (Wickham, 2016) and patchwork (Pedersen, 2024). Reporting of this work followed the RECORD-Statement (Benchimol et al., 2015).

### 2.7 Sensitivity analyses

We conducted two sensitivity analyses on the staffing measure with the most significant downward trend, the supply-demand-match. First, we recalculated the supply-demand-match including both *direct* and *indirect care* time. Second, we accounted for a legislation change in the classification of care complexity: Per January 1st, 2022, twelve of the 36 resource utilization groups were assigned to a lower (1) or higher (11) care demand level. This legal adaption reflects the reality of increasing time needs with certain clinical complexities, but it poses the risk of artificially steep changes in our data. To account for this, we calculated the supply-demand-match using the previous care demand classification of each resource utilization group.

## 3 Ethical considerations

This study relies on staff roster and insurance claims data and does thus not analyze any personal health data. The ethical committee of the canton of the study site ruled that the research project does not fall under the scope of the Swiss Human Research Act (HRA), because it is not defined as research concerning human diseases or structure and function of the human body as per the HRA Art. 2 para. 1 (BASEC Nr. Req 2021-00574). All data was pseudonymized prior to analysis.

## 4 Results

### 4.1 Study data and population

Across the study timeframe from January 1st, 2018 to December 31st 2023, we analyzed a total of 71,484,262 supply minutes delivered as direct care (+6502,006 additional minutes in leadership or supervisory positions) across 533,003 rostered staff shifts (of which 193,612 were delivered shifts), and 66,767,973 demand minutes across 387,585 resident days. Hereby we followed two open cohorts of 793 staff members and 1269 residents from seven long-term care units in a Swiss multisite long-term care organization. Five residents (0.4 %) and 89 staff members (10.3 %) were constantly attributed throughout the study duration (non-censored). A little more than half of residents are female (57.2 %), and the average age at entry into the organization is 80.6 years. The average care demand at admission is 7.4 (on a scale of 1- 2), indicating that a resident requires up to 151 min (∼2.5 h) of professional care time per day. The majority of staff members is female (87.1 %) and on average 34.2 years old.

Overall, the staff mix across the three staff groups in this study is 24.9 % RNs, 22.8 % LPNs and 52.3 % CNAs. Table 1 presents further information on baseline measures.

**Table 1:** Nursing home resident and staff characteristics at baseline.

Group	Characteristic	N ( %)	Mean (SD)
**Residents**		1269 (100.0)	
	Sex^a^ [female]	726 (57.2)	
	Age [years]		80.6 (10.2)
	Care demand level [scale: 1–12]^b^		7.36 (1.9)
	Total length of stay between 2018–2023 [days]		305.4 (504.2)
**Staff members**^c^^,^^d^		719 (100.0)	
	Sex [female]	626 (87.1)	
	Age [years]		34.2 (13.7)
	Staff tenure prior to study [years] (*n* = 254)		3.8 (5.0)
**Skill mix**^e^			
	Group 1-RNs	179 (24.9)	
	Group 2-LPNs	162 (22.5)	
	Group 3-CNAs (including students)	378 (52.6)	
	CNAs	212 (56.1)	
	Students/apprentices	166 (43.9)	

*Note.* Both are open cohorts across six years. As baseline characteristics we took measures from the first day of study or the first available record per person. RN = registered nurses, LPN = licensed practical nurses, CNA = certified nursing assistants.

a A binary representation of sex (female, male) is provided due to legislation in Switzerland at the time of study.

b Care demand level: A scale ranging from 1–12, with higher levels indicating higher care demand and more care time required by a resident.

c Includes permanently employed staff members only; 74 temporary staff members are unavailable for sample description as they are not listed in the organizational payroll system.

d Staff tenure was only calculated for staff members with tenure prior to the first day of study.

e Staff groups ordered by decreasing educational level (i.e., highest level = RNs). Group 1- RN includes *n* = 5 Physiotherapists; Group 2 - LPN includes *n* = 5 activation or art therapists; Group 3 includes also students/apprentices for further analysis, but is split for comparability in table.

### 4.2 How did staff supply meet resident demand?

**Fig. 1:**
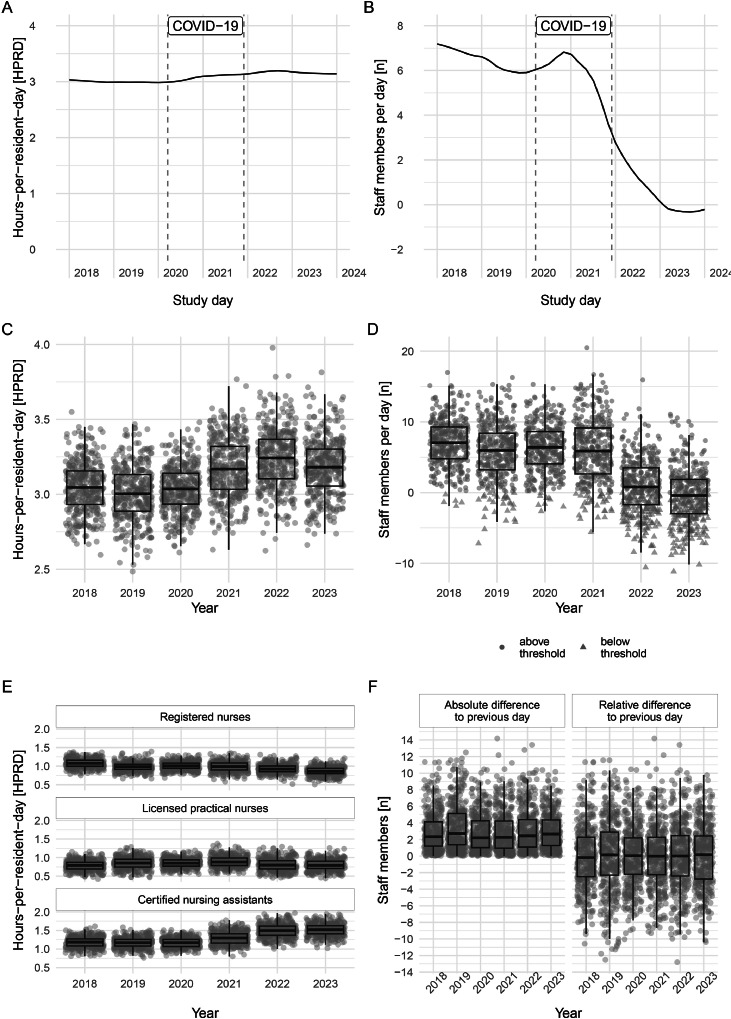
Daily trends (Panels A, B) and variation (Panels (C, D, E, F) for hours-per-resident-day (left column) and supply-demand-match (right column). The dashed vertical lines in Panels A, B represent the timeframe during which the organization operated under exceptional circumstances due to the pandemic.

Fig. 1 visualizes daily trends and variation of how staff supply met resident demand across the study duration by two different staffing measures, HPRD (Fig. 1A, C, E) and supply-demand-match (Fig. 1B, D, F) . Between 2018 and 2023, the daily facility level trends for total HPRD remained constant with a slight increase towards the end of 2021 (Fig. 1A). In contrast, the daily supply-demand-match adjusted by resident complexity steadily decreased on facility level dropping below the value 0 in 2023 (Fig. 1B). In numbers, the facility-level average direct care total HPRD ranged from 3.0 [95 % CI 3.0–3.1] in 2018 to 3.2 [95 % CI 3.2–3.2] in 2023 with RN—HPRD dropping from 1.07 [95 % CI 1.06–1.08] to 0.87 [95 % CI 0.86–0.88], LPN—HPRD remaining steady from 0.79 [95 % CI 0.79–0.80] to 0.80 [95 % CI 0.80–0.81] and CNA-HPRD increasing from 1.18 [95 % CI 1.17–1.19] to 1.52 [95 % CI 1.50–1.53]. Meanwhile, in the same samples, the yearly average supply-demand match decreased from +6.88 staff members [95 % CI 6.86–6.90] in 2018 to −0.23 [95 % CI −0.24 - −0.22] in 2023. This indicates that for the same resident case mix on average 1.19 fewer staff members per day were available during each study year. Both measures varied considerably between units. Yearly average measures are available in Supplementary Material B, Tables 1 and 2.

We further investigated daily operational variation of HPRD (Fig. 1C and E) and supply-demand-match (Fig. 1D and F). In brief, the within-group distribution for each fiscal year remains mostly constant (i.e., similar interquartile range IQR) within total HPRD (Fig. 1C), HPRD by staff group (Fig. 1E) and supply-demand-match (Fig. 1D). For example, with total HPRD the IQR (i.e., the difference between the 25th and the 75th percentile) increased from 0.22 (2018) to 0.25 (2023), which equals to as few as 1.8 min higher daily variation per resident day in half of the annual days in 2023 compared to 2018. However, between fiscal years the average operational measures change as previously illustrated by the trends. Most prominently, the supply-demand-match fell below the theoretical threshold of value 0 from 1.3 % days in 2018, to 55.1 % of days in 2023 (Fig. 1D). Interestingly, consecutive day variation – i.e., today’s supply-demand-match compared to yesterday – remained constant in absolute terms, but changes towards lower staffing became more extreme in 2023 (Fig. 1F).

### 4.3 Staff absences and deployment of temporary staff: determinants of and response to drop in supply-demand-match

Given the pointed decrease of supply-demand-match we investigated the suspected determinant of staff variation, i.e., daily number of staff absences, as well as the daily deployment of temporary such as pool or agency staff, which is a commonly used managerial strategy to overcome unplanned staff shortages. Across all *n* = 193,612 worked staff shifts the majority was worked by staff members on the focal unit they belong to (92.3 %). Despite an overall small remainder of shifts worked by other staff members, such as from an internal pool or external agency, this number increased from 5.5 % 2018 to 17.5 % in 2023.

**Fig. 2:**
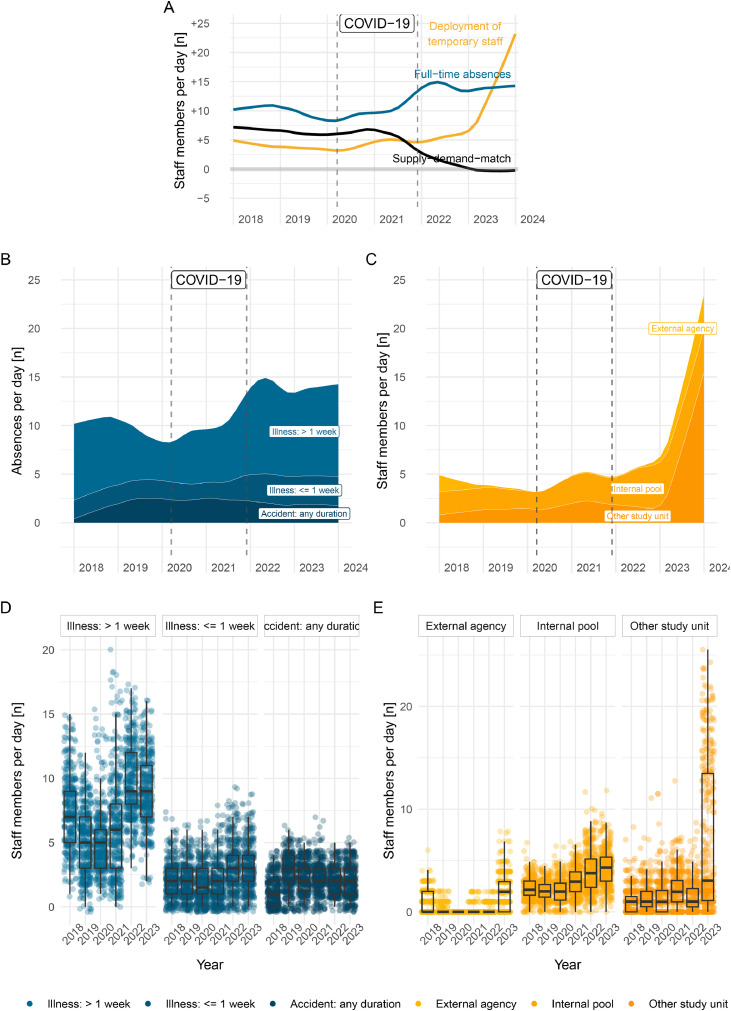
Contrasting trends for supply-demand-match, full-time absences and deployment of temporary staff across all study years (Panel A), trends (Panel B) and variation (Panel D) of absences by type, trends (Panel C) and variation (Panel E) of temporary staff by type. The dashed vertical lines in Panels A, B, C represent the timeframe during which the organization operated under exceptional circumstances due to the pandemic.

Fig. 2 contrasts the co-occurring trends (Panel A) as well as trends (B, C) and variation (D, E) between daily supply-demand-match with staff members absences and the deployment of pool or agency staff. Mid 2021 the increase in staff absences is aligned with the drop in supply-demand-match where both curves start diverging concurrently. In 2023, the spiking increase of pool or agency staff seems to soften the downward slope of the supply-demand-match to reach a steady trend (albeit on a low level) during the last study year (Fig. 2A).

In 2018, on average 11.09 [95 % CI 11.07–11.10] staff members per day had a full-time absence registered; this trend slightly decreased to 9.61 [95 % CI 9.52–9.70] in 2019 and 8.95 [95 % CI 8.90–9.00] in 2020. From 2021, the average daily absences increased to 11.32 [95 % CI 11.15–11.48] and spiked to 14.80 [95 % CI 14.73–14.88] in 2022 and remained at 14.23 [95 % CI 14.19–14.27] in 2023. During COVID-19 we observe an increase in illness-related absences of all durations. After the pandemic in 2023, illness related absences, especially durations ≥ 1 week, spike and remain on a constantly high level, while accidents slightly decreased (Fig. 2B and D). Concurrently, the daily number of shifts worked by temporary staff such as pool or agency staff started at 4.26 [95 % CI 4.22–4.29] in 2018 and decreased in 2019 to 3.59 [95 % CI 3.57–3.60] and 2020 to 3.67 [95 % CI 3.62–3.72], respectively. Between 2021 (4.84 [95 % CI 4.83–4.86]) and 2022 (5.51 [95 % CI 5.45–5.56]) an increase is forming, with an unparalleled spike in 2023 to 13.91 [95 % CI 13.40–14.43]. Despite this spike, the deployment of external agency staff represents only a small proportion of shifts in the whole study period (Fig. 2C and E); the majority of shifts are worked by internal pool staff or by staff members that belong to the facility but usually work on another unit.

### 4.4 Untangling the supply-demand-match: how did staff supply and resident demand evolve?

#### 4.4.1 Supply variation by staff mix and type of care

**Fig. 3:**
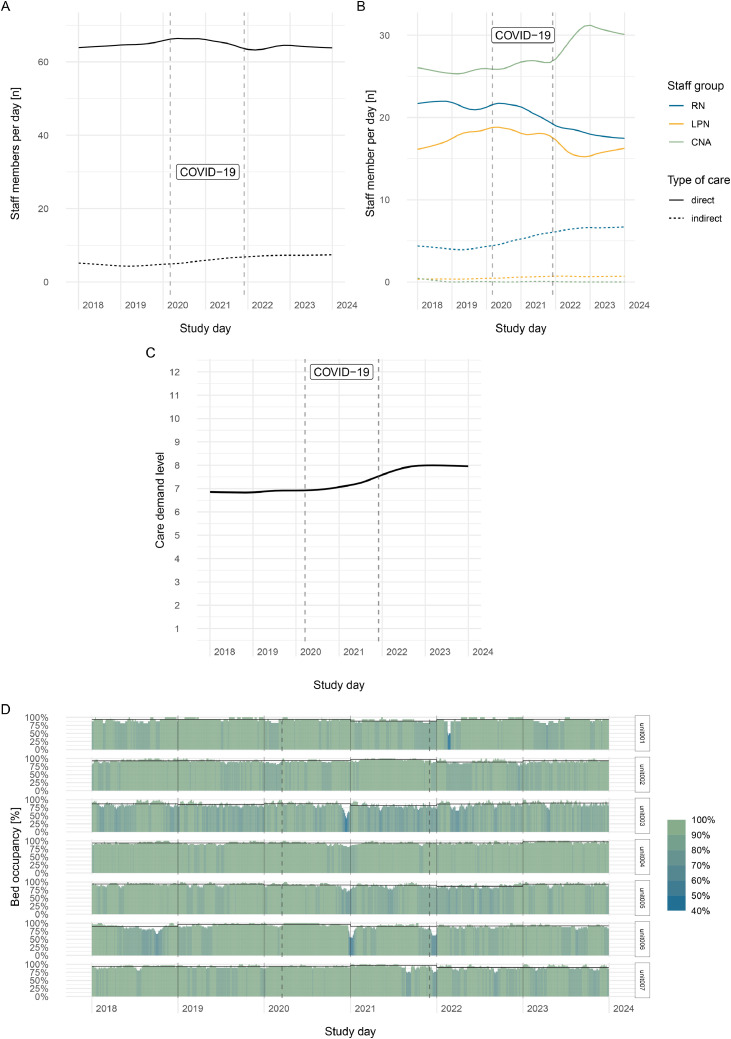
Comparing changes in supply trends and care delivery trends (Panel a, b) and demand characteristics by complexity (Panel c) and variation in bed occupancy (Panel d).

We visualized how staff supply changed, both by staff mix as well as type of care in (Fig. 3). The total staff time remained constant across the study timeline, (Fig. 3A) but staff mix changed with a decrease in direct care provided by higher-educated staff such as RNs and LPNs from 2021 and an increase in CNA’s time (Fig. 3B). Total direct-care staffing time slightly decreased and indirect care (i.e., staff in managerial or clinical supervisory or leadership roles) increased slightly across the study time.

#### 4.4.2 Resident demand by census and care complexity

Lastly, we investigated the changes in resident demand. As evident from Fig. 3c, the facility level average care demand trends increased from 6.83 [95 % CI 6.83 – 6.85] in 2018 to 7.99 [95 % CI 7.97 - 8.01] in 2023, whereas the unit level average care demand level ranged from 6.33 [95 % CI 6.29 - 6.37] to 9.32 [95 % CI 9.27 - 9.37] across all years [Supplementary Material B, Table 3].

Between 2018 and 2023, resident census remained constant within units with a median unit size of 28 beds (range: 17–33). The average yearly proportional bed occupancy per unit ranged from 81.3 % to 96.9 %, with occasional short-time operational variation (Fig. 3, Panel D). While we observed a general seasonal effect with lowest census in November, there was no demand variation based on weekdays aligning with the residential situation in a long-term care facility.

## 5 Results from sensitivity analysis

**Fig. 4:**
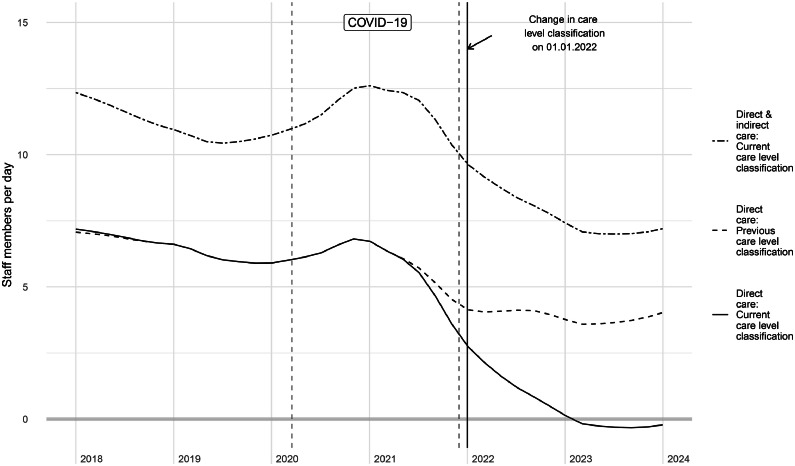
Sensitivity analysis: comparison of different scenarios for supply-demand-match. The solid line represents the main outcome reported in previous figures.

We compared the supply-demand-match (direct care with current care demand level-classification), with two alternative scenarios: 1) match calculated including both direct and indirect care with current care demand level-classification, 2) match including direct care only, but with previously applied care demand level-classification. Our sensitivity analysis confirmed the drop in supply-demand-match in both scenarios, albeit less pronounced as shown in Fig. 4.

## 6 Discussion

Our study provides a detailed analysis of care staffing patterns over a six-year period on both facility and unit level within a large multisite long-term care organization in Switzerland. From 2018 to 2023, the imminent workforce scarcity is brought to the surface with a facility-level supply-demand-match that consistently decreased before and after COVID-19. With constant variation within fiscal years, but decreasing trends across time, this indicates an overall lower staff availability for the same case mix. In addition to an increase of care complexity levels on the demand side, the decrease was concurrently observed to a high number of staff absences. These absences rose notably from 2020 and could not be mitigated fully despite managerial responses such as increased deployment of staff from other units, the internal pool, and external agencies. However, these necessary adjustments in supply resulted in a shift of care delivery, characterized by a lower staff mix providing direct resident care and a higher staff mix in indirect (supervisory) positions. These results have implications on multiple levels.

The persistent rise of staff absences indicates that the pandemic's effects on workforce availability were more profound and enduring than initially anticipated (Brazier et al., 2023; McGilton et al., 2020). Previous research has demonstrated the impact of COVID-19 on increased sickness rates among healthcare workers during the pandemic, attributable to both personal infection and also precausory measures to prevent nosocomial infection (Reme et al., 2023). Interestingly, in our sample, and in contrast to international research, the staff shortage was less pronounced during COVID-19 (Yang et al., 2021), but increased subsequently, particularly after the formally announced termination of the pandemic situation in Switzerland. Part of this spike can be attributed to the reduction of mask-wearing policies and other. Still, we observe an increase in absences with duration of longer than 1 week compared to pre-pandemic trends, that remained on high levels until the end of the follow-up period. In Switzerland, legally, every absence exceeding 3 days needs to be signed off by a medical doctor (Herzog-Zwitter et al., 2021), thus absences exceeding 7 days suggest higher illness severity including a personal doctor’s visit. We hypothesize an interplay of several contributing mechanisms: First, the increased potential for post-acute sick leave following a COVID-19 infection, which has been discussed as a high societal burden in the general population (O’Regan et al., 2023), also applies to care staff. Second, earlier work has repeatedly demonstrated the link between high individual workload and burnout (Diehl et al., 2021), with burnout being a relevant antecedent for absences and absenteeism among health care workers (Lee et al., 2023). In our study, this vicious circle of absences and higher individual workload appears from mid 2020, with the severe drop in direct care staff availability. This is supported by our sensitivity analysis, which suggests indirect care workers are less affected by absences, which could hint at both lower levels of burnout in non-frontline staff but also at higher levels of presenteeism (in case of light illness) as was reported elsewhere for indirect care positions (Linsenmeyer et al., 2023). Regardless of their cause, the quantity of absences imposes both a high financial burden and impacts on the remaining staff.

We observed a clinically significant reduction in available workforce with an average decrease of more than 1 staff member each year, given the same case mix. To counteract the downward trend, the organization reacted with increased deployment of pool and agency staff. Previous research has demonstrated the link of temporary staff to lower staff stability and higher staff turnover, as described elsewhere (Ward et al., 2001). In our sample, most temporary staff were attributed to the organization (i.e., re-deployment from other unit or internal pool staff), and thus we did not investigate turnover as a structural measure. However, concurrent with the increased deployment of temporary staff, we observed a decline of skill mix in direct care, indicating that lower staffing levels were utilized for replacements. While we cannot rule out an organizational strategy, we suspect this was also influenced by job market availability, as a pre-pandemic survey study with 118 Swiss long-term care organizations revealed recruitment difficulties for RNs and LPNs (Zúñiga et al., 2021). Regardless of the factors driving this shift in skill mix, from an organizational perspective, an adaptation in care model delivery based on the scope of practice of the involved staff members is necessary. Earlier research examined the relationship of skill mix with resident outcomes (Yang et al., 2020), and as well as the use of agency staff and its impact on quality of care (Castle and Engberg, 2008; Ma et al., 2023). Based on existing evidence there is a potential risk of reduced quality of care, which warrants further exploration through longitudinal research.

Our study presents two complementary analytical perspectives on the availability of staffing in Switzerland. While the trends for HPRD remained constant throughout the study duration, the case-mix adjusted supply-demand-match steadily declined for the same samples, falling below zero on more than 50 % of days in the last study year. The divergence in observed trends between both measures is a reflection of the increasing care complexity of residents, which we see both in our study as in other research (Ng et al., 2020). Based on our analysis, we do not recommend on using HPRD as a sole staffing measure in both research or workforce policies, aligning with previous recommendations (Mukamel et al., 2023).

The decline of staff availability measured by supply-demand-match is likely noticeable in daily practice, yet, we urge caution in the interpretation of the theoretical threshold of value zero for actual staff shortage: Several studies on care time indicate that resident demand is frequently underestimated (Brühl et al., 2018). As care demand often exceeds the maximum reimbursed daily care time of 240 min or with an increase in tasks not covered by resident utility groups, it is possible that the theoretical threshold of value zero is set too low. In addition, in the absence of staffing regulations in Switzerland, interpretation of a possible impact on resident outcomes remains open. Regardless of the type of staffing measure, it is worthwhile to note the high between-unit variation of both HPRD and supply-demand-match. This has potential implications for association analyses between staff and outcome data from facility level-data sources as mentioned, as aggregated data has the potential to mask nuanced findings (Greenland, 2001; Winter et al., 2021).

### 6.1 Strengths and limitations

To our knowledge, this is the first study to comprehensively analyze operational staffing patterns using data from a multisite provider in Swiss long-term care. The application of time-series decomposition instead of longitudinal aggregation allowed us to account for effects in seasonality while isolating temporal trends, thereby retaining the daily granularity (Cleveland et al., 1990). Despite the large sample size and the effective mitigation of endogeneity present in large scale provider comparisons, our single-center approach limits unconditional generalizability of our findings to other long-term care organizations, both internationally and within Switzerland. Nevertheless, given the paucity of published granular staffing analysis for Swiss long-term care, our study provides valuable insights and serves as a foundation for further analysis and policy dialogue. The descriptive analysis provides insightful perspectives on current quantitative care staffing trends; however, it does not yet permit to draw any conclusions on how these measures have impacted resident outcomes, necessitating further research. Finally, a limitation inherent to the use of administrative data is the omission of qualitative staffing measures, such as staff attitude, which can influence teamwork and consequentially, affect the resident-staff-relationship as well as residents’ satisfaction and quality of life.

### 6.2 Conclusions

Although our study indicates adherence to a theoretical staffing threshold, it also reveals a consistent decrease of nursing home care staff availability over the past six years. This decline persisted despite targeted organizational efforts to mitigate it. Our findings underscore relevant aspects gained by operational staffing levels and suggest nuanced changes over time concerning lower skill mix. With the previously unrecognized increase of longer co-worker absences – despite structured absence management system – our results suggest a complex interaction on top of existing challenges in employee recruitment retention to address for nursing home care workforce management. Our results have organizational implications, and demand an adaption of existing models of care delivery to ensure and maintain adequate care quality. Likely, these adaptions come with increased financial costs to an organization. Healthcare policies should consider alternative reimbursement and employment strategies to reduce the financial burden of organizations.

## Funding

This research did not receive any specific grant from funding agencies in the public, commercial, or not-for-profit sectors.

## CRediT authorship contribution statement

**Catherine Blatter:** Writing – review & editing, Writing – original draft, Visualization, Validation, Software, Resources, Project administration, Methodology, Investigation, Formal analysis, Data curation, Conceptualization. **Michael Simon:** Writing – review & editing, Validation, Supervision, Software, Methodology, Investigation, Formal analysis, Conceptualization. **Franziska Zúñiga:** Writing – review & editing, Validation, Supervision, Resources, Project administration, Investigation, Conceptualization.

## Declaration of competing interest

The authors declare that they have no known competing financial interests or personal relationships that could have appeared to influence the work reported in this paper.

## Data Availability

The raw data underlying this study are not publicly available. Code underlying this analysis or further tabulated results for staffing measures are available from the author upon reasonable request.

## Appendix Supplementary materials

Image, application 1

Image, application 2

Image, application 3
