# Supplementary figures and images for: Daily staffing trends and variation in Swiss long-term care from 2018–2023: A retrospective longitudinal analysis
Source: Int J Nurs Stud Adv. 2025 Jul 29;9:100395. doi: 10.1016/j.ijnsa.2025.100395 (PMC12801158; doi:10.1016/j.ijnsa.2025.100395)

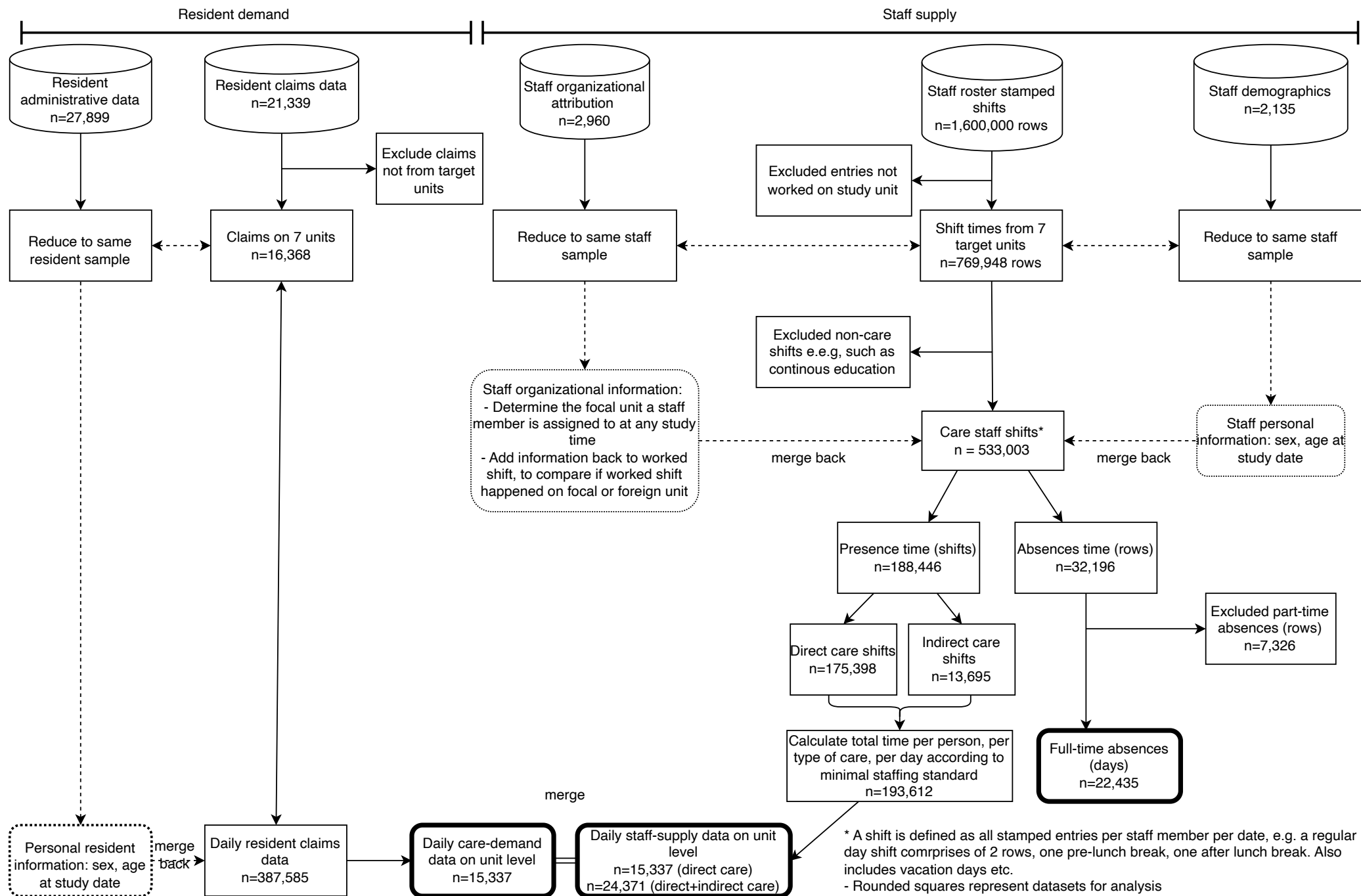

Supplement: Supplementary file 1 [file mmc1.pdf]
