# Supplementary material for: Daily staffing trends and variation in Swiss long-term care from 2018–2023: A retrospective longitudinal analysis
Source: Int J Nurs Stud Adv. 2025 Jul 29;9:100395. doi: 10.1016/j.ijnsa.2025.100395 (PMC12801158; doi:10.1016/j.ijnsa.2025.100395)
Supplement: Supplementary file 3 [file mmc3.docx]

**Supplementary material B**

**Supply characteristics**

Table 1. Yearly average HPRD (direct care only)

|  | 2018 | 2019 | 2020 | 2021 | 2022 | 2023 |
| --- | --- | --- | --- | --- | --- | --- |
| RN | 1.03 | 0.96 | 0.99 | 0.97 | 0.93 | 0.88 |
| LPN | 0.79 | 0.84 | 0.86 | 0.89 | 0.80 | 0.80 |
| NA | 1.20 | 1.18 | 1.18 | 1.29 | 1.48 | 1.51 |
| Total | 3.01 | 2.98 | 3.01 | 3.13 | 3.18 | 3.15 |

**Indirect and direct care**

Table 2. Yearly average HPRD (direct and indirect care)

|  | 2018 | 2019 | 2020 | 2021 | 2022 | 2023 |
| --- | --- | --- | --- | --- | --- | --- |
| RN | 1.23 | 1.15 | 1.2 | 1.25 | 1.25 | 1.19 |
| LPN | 0.81 | 0.86 | 0.88 | 0.92 | 0.83 | 0.83 |
| NA | 1.21 | 1.18 | 1.18 | 1.30 | 1.48 | 1.51 |
| Total | 3.23 | 3.18 | 3.25 | 3.45 | 3.54 | 3.50 |

**Demand characteristics**

Table 3. Yearly unit-level measures of average care demand level per unit

| Unit | 2018  *Mean* [95% CI] | 2019  *Mean* [95% CI] | 2020  *Mean* [95% CI] | 2021  *Mean* [95% CI] | 2022  *Mean* [95% CI] | 2023  *Mean* [95% CI] |
| --- | --- | --- | --- | --- | --- | --- |
| unit001 | 6.92 [6.86-6.98] | 7.45 [7.39-7.50] | 7.58 [7.53-7.63] | 7.46 [7.41-7.51] | 9.25 [9.20-9.30] | 9.32 [9.27-9.37] |
| unit002 | 7.00 [6.97-7.04] | 6.80 [6.77-6.84] | 6.62 [6.59-6.65] | 6.83 [6.79-6.86] | 7.17 [7.12-7.22] | 7.53 [7.48-7.58] |
| unit003 | 7.27 [7.25-7.30] | 7.45 [7.42-7.48] | 6.68 [6.64-6.72] | 7.01 [6.97-7.06] | 7.68 [7.64-7.73] | 7.61 [7.56-7.65] |
| unit004 | 6.51 [6.46-6.55] | 6.54 [6.50-6.58] | 6.86 [6.80-6.91] | 7.11 [7.06-7.16] | 7.55 [7.50-7.59] | 7.16 [7.11-7.20] |
| unit005 | 6.33 [6.29-6.37] | 6.59 [6.55-6.63] | 7.01 [6.96-7.05] | 7.16 [7.11-7.21] | 7.95 [7.90-8.01] | 8.10 [8.05-8.16] |
| unit006 | 7.48 [7.45-7.52] | 6.89 [6.86-6.92] | 6.53 [6.49-6.56] | 6.84 [6.80-6.88] | 7.45 [7.41-7.49] | 8.36 [8.32-8.40] |
| unit007 | 6.46 [6.42-6.50] | 6.92 [6.88-6.96] | 7.55 [7.52-7.59] | 8.15 [8.12-8.17] | 8.76 [8.73-8.80] | 8.49 [8.44-8.53] |
